# The invisible 800-pound gorilla: expertise can increase inattentional blindness

**DOI:** 10.1186/s41235-023-00486-x

**Published:** 2023-05-29

**Authors:** Samuel G. Robson, Jason M. Tangen

**Affiliations:** 1 School of Psychology, The University of Queensland, St Lucia, QLD 4072 Australia; 2 School of Psychology, The University of New South Wales, Kensington, NSW 2052 Australia

**Keywords:** Inattentional blindness, Perceptual expertise, Visual attention, Fingerprint identification

## Abstract

People can fail to notice objects and events in their visual environment when their attention is engaged elsewhere. This phenomenon is known as inattentional blindness, and its consequences can be costly for important real-world decisions. However, not noticing certain visual information could also signal expertise in a domain. In this study, we compared professional fingerprint analysts and novices on a fingerprint matching task in which we covertly placed an image of a gorilla into one of the prints. This gorilla was either small, or large, but always embedded in a way that made it largely irrelevant to the primary task. We found that analysts were more likely than the novices to miss the large gorilla. We interpret this finding not as a flaw in how these experts make decisions, but most likely an expression of their expertise; instead of processing more information they filter out irrelevant information and constrain their attention to what is important.

## Introduction

People regularly fail to notice objects in their visual environment when their attention is directed elsewhere even when these objects appear obvious in retrospect. This phenomenon is known as inattentional blindness (Mack & Rock, 1998). In the best-known demonstration of this effect, Simons and Chabris (1999) instructed participants to count the number of times a basketball team passed a ball to one another. Most people could accurately count the passes, but many failed to notice a person dressed as a gorilla walk through the scene. This is a memorable demonstration of how conspicuous objects can go unnoticed because people have a finite attentional capacity.

Expertise in a domain, however, may lower how susceptible a person is to inattentional blindness (Ekelund et al., 2022; Memmert, 2006; Simons & Schlosser, 2017). Skilled basketball players who viewed the video described above, for example, were more likely than novice counterparts to notice the gorilla (Memmert, 2006). Medical diagnosticians, though not immune to inattentional blindness (see Lum, et al., 2005; Williams et al., 2021), are less susceptible than non-diagnosticians during radiological tasks. Drew et al. (2013) instructed medical experts and novices to locate suspicious lung nodules in a stack of Computed Tomography slides. Unbeknownst to participants, an image of a gorilla was inserted into a handful of the slides. Only four of the 24 radiologists noticed the gorilla, whereas none of the novices noticed.

In the present study, we further investigate the effect of expertise on inattentional blindness using a similar paradigm to Drew et al. (2013), but with fingerprint analysis as the case domain. The job of a fingerprint analyst is to decide whether two different fingerprint impressions came from the same source. These analysts demonstrate genuine expertise across a range of domain-specific perceptual tasks (Busey & Parada, 2010; Searston & Tangen, 2017a, 2017b; Tangen et al., 2011; Thompson & Tangen, 2014; Thompson et al., 2014; Vogelsang et al., 2017). In the present study, fingerprint analysts and novices complete a fingerprint comparison task, but on the last trial, an image of a gorilla is embedded in one of the fingerprints. Whereas Drew and colleagues positioned a small gorilla near a lung nodule, which is a *task-relevant* feature, we place the gorilla so that it resembles information that is *irrelevant* when matching prints. In one condition, we position a small gorilla in an area of smudging or unremarkable ridge detail somewhere in the periphery of a fingerprint (local condition). In another condition, the silhouette of a gorilla is blended into a large section of a fingerprint in the form of thickened ridges (global condition).

There is reason to believe that fingerprint identification performance, as with performance in diagnostic medicine, may be affected by inattentional blindness. Some evidence suggests that missing certain minutiae in a fingerprint can negatively affect identification performance (Loyola-González et al., 2021). The aim of the present study, however, is not to measure the rate of inattentional blindness in fingerprint analysts, nor how inattentional blindness affects matching performance. Rather, our aim is to understand whether the relationship between expertise and inattentional blindness depends on the nature of unexpected object.

We have three competing hypotheses. One prevailing theory for why experts may be less inattentive than novices to unexpected objects is that experts are less occupied by the primary task (see Drew et al., 2013; Fougnie & Marois, 2007; Richards et al., 2010; Simons & Jensen, 2009). As one gains experience, familiar tasks become effortless, freeing up mental resources, and leaving one more receptive to unexpected events. Even when time is limited, fingerprint analysts outperform novices at matching prints (e.g., Thompson & Tangen, 2014), indicating that fingerprint comparisons are easier for professional analysts than for novices. If more experts than novices notice the gorilla in the print, this would suggest that experts are more likely to notice the unexpected gorilla since they devote less attention to the primary task (Hypothesis 1).

Alternatively, experts may notice the gorilla less frequently than novices because it has been integrated into task-irrelevant visual detail, and this may also depend on a local versus global attentional focus. With practice, a person becomes sensitive to the relevant features and dimensions within their domain (Goldstone, 1998; Kellman & Garrigan, 2009). Over time, fingerprint analysts become attuned to the features that are most diagnostic for telling fingerprints apart. Compared to novices, for example, professional analysts focus on fewer but more task-relevant features when examining prints (Busey & Parada, 2010; Roads et al., 2016; Robson et al., 2020). Formal fingerprint training guidelines also emphasise a slow, analytic comparison of ridge detail and minutiae (SWGFAST, 2012). As such, when comparing fingerprints, fewer analysts than novices may detect a small gorilla that has been placed in a relatively unimportant localised area (Hypothesis 2).

Other evidence indicates that fingerprint analysts process prints holistically, focusing their attention on diagnostic global information, such as the overall pattern type, configuration, or general ‘style’ of the print (see Busey & Vanderkolk, 2005; Searston & Tangen, 2017b). Indeed, holistic processing is a hallmark of visual expertise in many domains (Curby et al., 2009; Diamond & Carey, 1986; Richler et al., 2009; Searston & Tangen, 2017b; Thompson & Tangen, 2014; Vogelsang et al., 2017). Analysts may therefore disregard irrelevant information that is distributed across a fingerprint—such as surface type and ridge thickness—including a gorilla if it was embedded within this information. In the global condition, where the gorilla is blended across a large section of the print, we may detect more inattention from experts than from novices (Hypothesis 3).

## Method

### Preregistration

All inclusion criteria, methods, hypotheses, and planned analyses were preregistered on the Open Science Framework (OSF) prior to collecting data. The preregistered project can be found here: https://osf.io/rtm58. We withdrew the preregistration as it contained materials that we cannot make available, but the project wiki explains the hypotheses and analysis plan time-stamped prior to collecting data.

### Participants

We tested 40 professional fingerprint analysts (*M*_age_ = 43.0, *SD* = 8.16; 19 females, 21 males) from various forensic agencies across Australia. This group included 30 accredited, court-practicing analysts, and 10 trainee analysts with at least one year of training (*M*_exp_ = 10.1 years, *SD* = 6.29; Range: 1–24 years). We also collected data from 40 novice participants (*M*_age_ = 21.7, *SD* = 5.59; 30 females, 10 males) from The University of Queensland community. Novice participants received either one course credit or the equivalent of 10 Australian dollars for their time. This sample size was sufficient (> 0.8 power) to detect a medium-to-large effect (*w* = 0.45) in each condition.

### Design and procedure

We employed a between-subjects design in which participants were randomly allocated to one of two conditions (local or global). Participants first provided demographic information before watching several instructional videos and completing two practice think-aloud problems. For instance, they were prompted to think aloud while recalling the number of windows in their parents’ home. After the prompt disappeared, participants were instructed to describe what they could remember thinking as they solved the previous problem, and what they found most memorable or distinctive.

After these practice questions, participants began the fingerprint portion of the experiment. During each trial, they were presented with two fingerprints side-by-side and were prompted to think aloud as they decided whether the prints matched or not. Participants could look at the images as they pleased, and for as long as they pleased, to mimic natural viewing conditions. However, participants could not zoom in, rotate, or manipulate the images in any way. Once participants made a decision, the fingerprints disappeared. Participants were then asked to describe what they could remember thinking as they analysed the prints, including what features they found most memorable or distinctive.

**Fig. 1:**
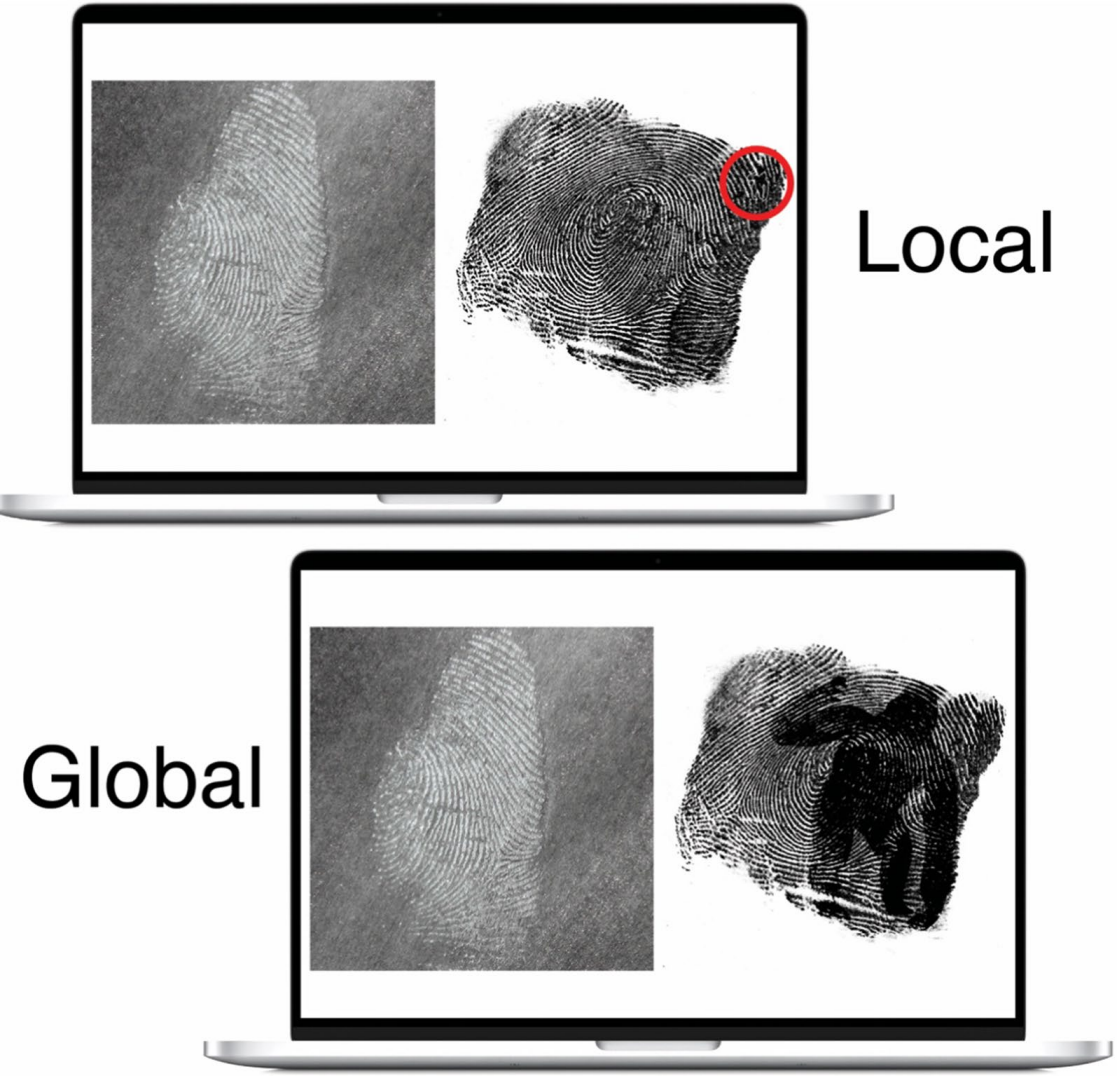
Example fingerprints used for the final comparison of the experiment. The local condition (top; gorilla highlighted with red circle) and the global condition (bottom). For each participant, one of 10 fingerprints containing a gorilla was sampled and paired randomly with either a distractor or target print. Each novice saw an identical fingerprint pair as a paired expert participant

In total, participants compared six print pairs. The first five trials became progressively more difficult. On the sixth trial, participants were presented with two fingerprints, except one had the silhouette of a gorilla embedded within it. In the local condition, this gorilla was relatively small and located in the outer edges of a fingerprint (see Fig. 1). In the global condition, the gorilla was much larger and spanned nearly the entire print. Each novice participant was presented with an identical set of images in the same order as one of the expert participants, but the image set for each expert-novice pair was randomly sampled. On each trial, we measured participants’ accuracy and response time, as well as whether they detected the gorilla on the final trial.

If participants mentioned a gorilla during the final trial, the experiment ended immediately after the last comparison. If not, they were asked up to three follow-up questions: “Did the final trial seem different to any of the other trials?”, “Did you notice anything unusual in the final trial?” and “Did you notice a gorilla in the final trial?”. These questions were designed to capture whether participants noticed the gorilla but chose not to mention it. If participants answered “yes” to any of these questions, and they could accurately describe the gorilla, the experiment ended, otherwise it ended after the final question.

### Materials

We sourced 60 fingerprint pairs that ranged in difficulty (easy, medium, difficult) for the first five comparisons. Half of the fingerprint pairs were from the same source, and half were from different sources. The easy pairs contained two fully rolled *tenprints* (inked prints rolled from nail to nail) whereas during the medium and difficult comparisons a *latent* (crime-scene) print was presented on the left and tenprint on the right. An expert collaborator on the project assisted by rating each pair of prints as easy, medium, or difficult, but did not participate in the experiment.

We sourced 10 additional tenprints and embedded a gorilla image into them. These modified tenprints were presented alongside a crime-scene print from the same or different sources. In total, we generated 20 different fingerprint pairs for each condition for the final comparison, and images from this pool were randomly sampled for each participant.

All materials were presented on 13-inch MacBook screens, which were set to 1400 × 900 pixels. All prints had a resolution of 700 × 700 pixels with dimensions of 14 × 14 cm when presented on screen. Each session was captured with two Zoom Q2n cameras and microphones.

#### Local gorilla fingerprints

To create the fingerprints for the local condition, we superimposed a silhouette of a gorilla onto each of the tenprints using Adobe Photoshop. We positioned the gorilla on the print’s periphery, in a region that was either smudged or had unremarkable ridge detail. Experts often consider areas of this sort to be less useful (see Robson et al., 2020). Similar to Drew et al. (2013), we also added a white outline around the gorilla. Moreover, we conducted a pilot experiment in which we gradually increased the size and opacity of the gorilla during a fingerprint comparison task (see this project). In the present experiment, we used the level of concealment at which roughly 50% of pilot participants noticed the gorilla, allowing us to compare experts and novices with the greatest sensitivity. The height of the local gorilla on screen was 1 cm.

#### Global gorilla fingerprints

To create the images for the global condition, we superimposed a silhouette of a gorilla onto each of the ten fingerprints and adjusted the gorilla’s size to fit within the print’s boundaries. We then blended it into the print using Adobe Photoshop such that the gorilla’s form appeared to be shaped by thickened ridges or added pressure—variability that analysts are normally trained to tolerate because pressure varies widely from impression to impression (Vanderkolk, 2011). The gorilla’s opacity was determined using a similar piloting procedure as that used for the local stimuli.

## Results

### Detecting the gorilla

**Fig. 2:**
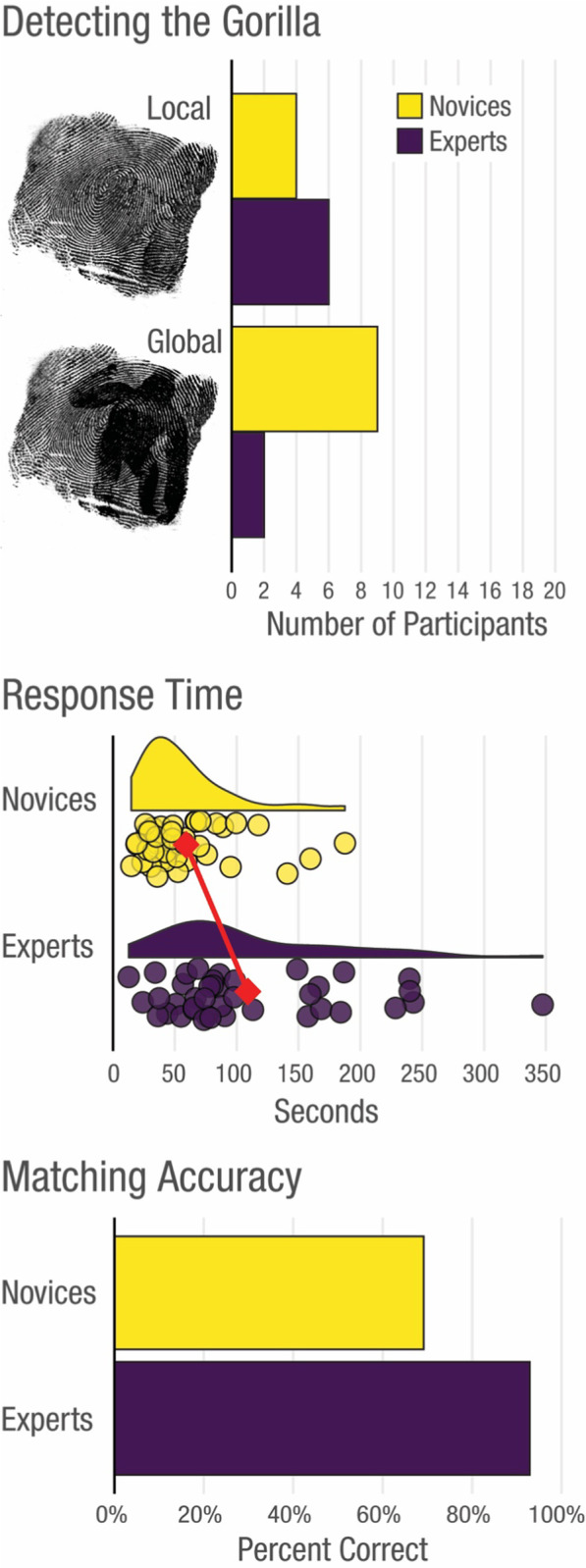
The number of novice and expert participants who detected the local and global gorilla (top). The number of seconds to respond during the final trial for each group with a red line connecting the group means (middle). The accuracy of each group averaged across all six fingerprint comparisons (bottom)

The primary finding in this experiment was that there was no significant difference in detection between novices and experts for the local gorilla, but more novices than experts spotted the global gorilla (see Fig. 2). Four of the 20 novices (20%) and six of the 20 experts (30%) detected the local gorilla, and this group difference was non-significant, *χ*^2^ (1, *N* = 40) = 0.53, *p* = 0.468. However, in the global condition, nine of the 20 novices (45%) detected the global gorilla, which spanned most of the print compared to only two of the 20 experts (10%), and this group difference was statistically significant, *χ*^2^ (1, *N* = 40) = 5.26, *p* = 0.022. An exploratory binomial logistic regression where we included data from experts and novices in both the local and global conditions revealed an interaction between Expertise and Condition, *b* = −0.63, *p* = 0.027. This result suggests that the expert-novice difference in the global condition was statistically different from the expert-novice difference in the local condition.

Note that these analyses are based on lenient criteria where a detection included mentioning the gorilla during the final trial or in response to any of the follow-up questions. The pattern of results was similar even when we did not include the final prompt (medium criteria). However, if detections are coded only as noticing the gorilla during the critical trial itself (strict criteria), then the group difference in neither condition is significant. Nevertheless, all participants who claimed to have noticed the gorilla after being prompted by a follow-up question were able to accurately describe the gorilla’s location and appearance in hindsight. We therefore think that the lenient criteria best reflect whether participants noticed the gorilla.

### Response time

An exploratory analysis of response time during the trial in which the gorilla appeared in the fingerprint revealed that experts spent longer (*M* = 108.0 s, *SD* = 60.6 s) than novices (*M* = 54.6 s, *SD* = 29.3 s) deciding whether fingerprints matched or not, *W* = 1198, *p* < 0.001 (see Fig. 2). Expert analysts were thus less likely to notice the global gorilla despite spending more time than novices analysing the fingerprints.

### Proportion correct

We compared the matching accuracy of experts and novices across all trials to determine whether experts found the primary task easier than novices. Overall, experts (*M* = 0.93, *SD* = 0.11) outperformed novices (*M* = 0.69, *SD* = 0.20), *W* = 1336, *p* < 0.001. Experts made no false alarm errors compared to 24.6% for novices, whereas experts made 14.2% misses compared to 34.2% for novices. This pattern of performance is similar to previous studies of fingerprint experts in which experts outperform novices, but also have a conservative response bias (e.g., Tangen et al., 2011).

## Discussion

We investigated the effect of expertise on inattentional blindness using fingerprint analysis as a case domain in this study. Experts and novices took part in a fingerprint comparison task, but on the final trial a gorilla was present in one of the prints. We compared how many participants from each group noticed the gorilla.

Prior studies have demonstrated that domain experts are more receptive than novices to unexpected objects because experts find the primary task easier, leaving them more receptive to unexpected events and objects (Drew et al., 2013; Fougnie & Marois, 2007; Memmert, 2006). However, in the present study, we placed a gorilla image within information that was irrelevant to the primary task and embedded it either locally or globally. We found no significant difference in detection between experts and novices for the local gorilla. In line with Hypothesis 3, however, experts were less likely than novices to notice the global gorilla that spanned nearly the entire print. Moreover, the experts performed better on the primary matching task, and spent longer analysing the fingerprint pair that contained the gorilla. Domain expertise therefore does not always inoculate observers against inattentional blindness. More inattention under certain circumstances may actually signal genuine expertise.

Over time, experts typically become sensitive to the features that best discriminate categories in their domain (Goldstone, 1998; Kellman & Garrigan, 2009), which may explain why fingerprint analysts and novices differ in the features they attend to (Roads et al., 2016; Robson et al., 2020). In the global condition, the gorilla was embedded so that it resembled thickened ridges, smudging or over-inking. Changes in pressure, and imperfections when fingerprints are deposited, are common sources of within-fingerprint variability (see Vanderkolk, 2011), which experienced analysts may learn to ‘see through’ with training. Thus, many experts may have missed the global gorilla because it represents visual information that is incongruent with what they typically pay attention to (see Ho et al., 2017 for a similar finding in medicine). This idea is related to the notion of an observer’s attentional set (Most et al., 2001, 2005; Pammer & Blink, 2013), which is a bias toward restricted perceptual aspects in the environment (Most, 2013). Experts may have a different attentional set than novices and this may affect what objects they are likely to notice or miss.

How might experts’ attentional set explain the higher rates of inattentional blindness in the global condition? We offer two possibilities. Fingerprint experts may have a narrower attentional window than novices given the emphasis of minutiae in their training (Busey et al., 2021) and their sensitivity to important, local features (Robson et al., 2021). If they are largely attending to local ridge detail, then this may ‘blind’ them to the global gorilla. Alternatively, their attention may be globally oriented, which is possible given evidence that they can process fingerprints holistically or non-analytically (Busey & Vanderkolk, 2005; Searston & Tangen, 2017b; Thompson & Tangen, 2014; Vogelsang et al., 2017). However, top-down knowledge and sensitivity to relevant visual structure may mean they see through irrelevant global information such as pressure or over-inking. Incidentally, these same processes may contribute to their superior matching performance relative to novices (see Tangen et al., 2011). More research is needed to investigate the interaction between local/global attention and relevant/irrelevant variability in the context of inattentional blindness.

This study has several limitations and alternative explanations. It is possible that the experts were more hesitant than novices to mention something as bizarre as gorilla when making their comparison. However, we included several follow-up questions to ensure we captured even those who noticed but did not feel comfortable saying so in the trial itself. Alternatively, the experts may have been more motivated to perform well on the primary matching task, leading them to focus more intensely on the task whereas novices may have taken this task less seriously, leaving them more receptive to unexpected objects such as a gorilla. However, a motivation to focus intensely on the task at hand could be viewed as a consequence of years of training and therefore an aspect of expertise. Third, expert and novice participants were not age-matched, and this may be a confounding variable given prior studies showing that inattentional blindness can increase with age (Graham & Burke, 2011). In any case, if general differences in motivation or age underlie our results, then the expert-novice difference ought to be the same direction for both the local and global conditions, but this was not what we found. An exploratory interaction instead revealed that the group difference for the global condition was different to the local condition.

Relatedly, there may be several reasons for why there was a non-significant group difference for the local condition. Perhaps both groups found the areas where we placed the gorilla to be irrelevant, so neither group paid much attention to these regions. The effect of expertise on inattentional blindness appears to depend heavily on the nature of the unexpected object. Several other studies, for instance, have failed to find significant differences in inattention between experts and novices (e.g., Ekelund et al., 2022; Memmert et al., 2009; Näsholm et al., 2014; Pammer & Blink, 2018).

In this study, we have demonstrated that experts can be more inattentive than novices to certain unexpected objects. However, this is not a reason to doubt the expertise of fingerprint analysts. Fingerprint analysts are highly competent at matching fingerprints, and their lower detection rate appears to have resulted *because* of their expertise rather than despite it. Those with considerable training in a perceptual domain like fingerprint analysis learn to attend to the most diagnostic perceptual information, and to ignore unimportant detail. Failing to notice a gorilla in a fingerprint is likely a manifestation of their learned inattention to irrelevant information.

## Data Availability

All de-identified data and analysis scripts can be found in the ‘Data and Analysis’ component of this OSF project: https://osf.io/rtm58. The experimental code and the images used in the critical trial of this study can be found in the ‘Experiment and Materials’ section of the project.
